# Ongoing challenges in access to diabetes care among the indigenous population: perspectives of individuals living in rural Guatemala

**DOI:** 10.1186/s12939-019-1086-z

**Published:** 2019-11-21

**Authors:** Edwin Nieblas-Bedolla, Kent D. W. Bream, Allison Rollins, Frances K. Barg

**Affiliations:** 1 Perelman School of Medicine, University of Pennsylvania, Philadelphia, PA USA; 2 Center for Global Health, Perelman School of Medicine, University of Pennsylvania, Philadelphia, PA USA; 3 School of Medicine, University of Washington, Seattle, WA USA; 4 Department of Family Medicine and Community Health, University of Pennsylvania, Philadelphia, PA USA; 5 Center for Public Health Initiatives, University of Pennsylvania, Philadelphia, PA USA

**Keywords:** Guatemala, Type 2 diabetes, Health inequities, Indigenous populations, Perceptions of disease, Access to care

## Abstract

**Background:**

Indigenous persons living in Latin America suffer from a higher prevalence of type 2 diabetes compared to their non-indigenous counterparts. This difference has been attributed to a wide range of factors. Future interventions could be influenced by a deeper understanding of the challenges that impact care in rural regions and in other low-income settings.

**Methods:**

This study was conducted using a modified grounded theory approach. Extended observations and fifteen interviews were performed with adult male and female residents of three rural Mayan towns in Sololá Department, Guatemala using purposive sampling. Questions focused on the perceptions of individuals living with type 2 diabetes and their caregivers regarding disease and treatment.

**Results:**

Across interviews the most common themes that emerged included mistreatment by healthcare providers, mental health comorbidity, and medication affordability. These perceptions were in part influenced by indigeneity, poverty, and/or gender.

**Conclusions:**

Both structural and cultural barriers continue to impact diabetes care for indigenous communities in rural Guatemala. The interviews in this study suggest that indigenous people experience mistrust in the health care system, unreliable access to care, and mental health comorbidity in the context of type 2 diabetes care. These experiences are shaped by the complex relationship among poverty, gender, and indigeneity in this region. Targeted interventions that are conscious of these factors may increase their chances of success when attempting to address similar health disparities in comparable populations.

## Background

Indigenous persons in Latin America suffer from worse health outcomes compared to their non-indigenous counterparts [1]. In Guatemala, over 40% of the population identifies as indigenous and experience similar health gaps as observed in indigenous populations of other countries [2, 3]. Type 2 diabetes disproportionately impacts indigenous communities in Guatemala. While the overall prevalence of type 2 diabetes and prediabetes is 9.3% in Guatemala, the prevalence amongst the indigenous community is 25% [4, 5] and is considerably higher than the prevalence of type 2 diabetes in Central America [5, 6]. A higher prevalence of type 2 diabetes has also been observed in other groups such as American Indian and Alaska Native populations, varying from 6.7% to nearly 22% [7].

In Guatemala, individuals receive type 2 diabetes care within a three-tiered healthcare system built to provide free health care to the population. These include hospitals, health posts, and health centers, all of which may provide varying levels of care to both indigenous and non-indigenous populations. Public hospitals are located throughout the region but may not be as accessible for those living in the country’s more rural areas [8]. Private, non-profit hospitals are also present, some of which have established outreach programs in collaboration with public health centers to provide type 2 diabetes care. Outreach can be inconsistent due to limited funding sources and transportation to larger hospitals can be challenging for those who live outside of major cities or towns. While public health centers are freely available and also provide services specifically targeted at type 2 diabetes screening, diagnosis, and management, they can be under-resourced [9].

Several factors may contribute to the increased risk of type 2 diabetes for indigenous populations, including changes in lifestyle and diet, healthcare underutilization, divergent etiology of disease, and unique genetic susceptibility [8, 10–12]. A recent switch to higher fat foods with the rise in availability of processed foods and a switch to a more sedentary lifestyle have led to an increase in obesity [13]. Healthcare utilization is far lower for indigenous populations due to sociocultural barriers, discrimination, and difficulty in accessing services. A study conducted in Sololá Department suggests that the necessities to provide reasonable care to the local indigenous population, including staff and adequate resources, is limited [8].

It has been well documented that indigenous communities are often subject to racial and discriminatory practices and in Latin America, social and political violence has been shown to inhibit access to quality care [14]. This is particularly true for Guatemalans, as a 36-years-long Civil War that ended in 1996 continues to have a powerful presence [15]. This war resulted in the massacre of indigenous people and the expropriation of their lands. A divergent cultural model for the etiology of disease is also present among the indigenous population of Guatemala that suggests type 2 diabetes emerges from certain life events or stresses, a concept that has been studied among Hispanic populations [16–19]. These studies have illustrated the broad beliefs that exist about the disease’s cause and progression such as the concept of *susto*, or that a traumatic life event or a “scare” could precipitate diabetes. Folk healers, or *curanderos*, are also present in these communities and may provide additional or alternative therapies for individuals with type 2 diabetes with unknown efficacy given the scarcity of studies evaluating these treatments [20]. The examination of possible underlying genetic dispositions of type 2 diabetes among indigenous populations remains an active area of study [21, 22]. The inequity of type 2 diabetes prevalence in Guatemala is multifactorial and will require broad and diverse efforts to resolve. There remains a gap in research when it comes to the experiences of indigenous individuals with type 2 diabetes in terms of their care despite the increasing prevalence of the disease amongst this group [8, 23]. Previous studies have focused on explanatory models or treatment practices used by some indigenous groups rather than the individual experience of illness. In this study we use a modified grounded theory approach to better understand how indigenous people experience living with type 2 diabetes and its management.

## Methods

### Study design

The study was conducted using a qualitative approach. One of the authors (ENB), who is a native Spanish speaker, spent approximately 3 months in Guatemala beginning in late May 2017. The majority of time was spent in the homes of two indigenous families in a rural town of Sololá Department, Guatemala; each household included at least one bilingual adult who spoke Spanish and the local Mayan language. The bilingual family member often served as an informal interpreter during interactions with non-Spanish speaking individuals. This translation helped both to clarify observations and allowed for more casual interviews to take place. The author was invited to participate in customs and traditions, was taught some of the local indigenous language, and a certain degree of confidentiality given private affairs was shared which allowed him to occupy the space of a relative insider. By living with indigenous families the author was able to participate in everyday activities with all members while establishing rapport and ties with the local indigenous community. Additionally, the author volunteered in the local community hospital and participated in several outreach programs, which included traveling to schools across the Sololá Department for educational presentations on diabetes and to remote areas of the region to provide diabetes screening and treatment.

An ethnographic inquiry shaped both data acquisition and analysis of this study. The author had the opportunity to observe interactions between health care professionals and members of multiple indigenous communities seeking diabetes care on an almost daily basis. Outreach programs presented further opportunities to interact as part of a healthcare team presenting a different perspective on how actions are intended and how this might differ from how they are perceived. In this context, the author participated both as an observer and active participant which ultimately influenced how future interviews were conducted and analyzed. These interactions allowed the author to appreciate the complex social, political, and economic reality in which individuals in this region live. Study of the cultural and historical background gave additional direction on how it might be possible to establish rapport, explore thoughts and relationships. This perspective allowed the interviewer to gain a greater understanding of the interactions that exist in the region between individuals and healthcare providers. The results from this study are compiled entirely from interviews with participants while the interpretation of these results have been enriched by both passive and active observation.

### Data collection

Data collection consisted of 15 semi-structured interviews and over 40 h of participant observations conducted across three municipalities – Sololá, Panajachel, and Santiago Atitlán, all in Sololá Department, Guatemala. Purposive sampling was used to enroll all participants. An interpreter native to the region and member of the local hospital staff was present for interviews not conducted in Spanish (*n* = 2). All interviews were carried out in July 2017. There was a concurrent analysis of data on site during this time in order to determine when saturation had been reached.

The exclusion and inclusion criteria included individuals over 18 years of age who reported having a diagnosis of type 2 diabetes from a health care professional or were a personal caregiver of someone with type 2 diabetes. All individuals included in this study had a permanent home in the region and self-identified as indigenous. We applied a pre-defined sampling frame specific for socio-demographic characteristics for sex and race because similar previous qualitative studies conducted in the area have tended to focus primarily on female participants when studying indigenous populations. We did not account for equal age distribution but did make attempts at obtaining participants from different age groups.

Verbal consent was obtained from all participating individuals. The purpose of the study was described as seeking to understand perceptions of type 2 diabetes and its care in the community. Individuals were also informed that there were no ‘right’ or ‘wrong’ answers and that all responses would be kept confidential despite being recorded. If any potential identifiers (names, dates, places, etc.) were mentioned, these were later to be redacted during the analysis. All questions to participants were intentionally broad in nature. Appropriate probing was used to elicit more information in relation to topics of interest, such as “You mentioned [topic of interest], can you tell me more about that?” In order to further abstain from influencing responses and to minimize chance of bias, individuals were reassured that any remaining questions they had about the project would be answered immediately following the interview.

A series of semi-structured interviews and participant observations were conducted in order to determine how social forces could be influencing diabetes care for indigenous communities in rural Guatemala. All interviews conducted in Spanish were transcribed verbatim from the participants’ responses. Interpreters were instructed to translate each word as faithfully as possible during interviews conducted in a language other than Spanish and these were later transcribed verbatim. All transcripts were translated to English by one of the authors (ENB) and verified for accuracy with an outside member that had no additional involvement in the study. Transcripts for these fifteen interviews are available in English and Spanish upon reasonable request.

### Analysis

A modified grounded theory approach was used for analysis [24]. Data analysis began simultaneously as interviews took place in order to inform future data collection. Point of saturation was determined after 15 interviews. No additional data was collected after identifying saturation. The transcripts were double-coded to assure consistency and prevent drift. First, two transcripts were used to identify broad categories that corresponded with outcomes of interest by applying hand-coding. This was done by one of the authors (ENB) and a second coder familiar with social science research and qualitative analysis. Joint examination of these transcripts then led to the creation of a draft codebook. The codebook was systematically updated and refined as appropriate throughout this time to catalogue all potentially relevant categories present in participants’ responses.

Next, emerging themes and sub-themes were developed in which no new concepts surfaced following successive coding confirming theoretical saturation. Themes and sub-themes were identified based on their relevance to the research objective, frequency within a transcript, and prevalence among responses. Inter-coder reliability based on double-coding between each set of independently coded transcripts was assessed through an iterative process using NVivo’s coding comparison query feature. The target Cohen’s Kappa coefficient value for each transcript was 0.75 or greater. The process was guided by another member of the team (FKB) with significant background in medical anthropology and qualitative research. Finally, interpretive findings were shared with staff of the local community hospital in Guatemala and others to critically discuss potentially varying interpretations of the data and increase validity.

## Results

There was a total of 15 participants. The interview sample was 53.3% (*n* = 8) female. The average age was 46.8 years old with a range of 22 to 71 years and a median of 47 years. Interviews were an average of 16 min in length with a range of 2 to 33 min. Of the 15 participants, six (40%) reported a previous diagnosis of type 2 diabetes while the remaining nine (60%) reported not having an official diagnosis or had not been recently tested but were caregivers for individuals with type 2 diabetes. Table 1 indicates the location were interviews were conducted.

**Table 1:** Location of Interviews in Sololá Department

Location	Number of Participants
Santiago Atitlán	2
Sololá	8
Panajachel	5

Interviews on the perceptions of type 2 diabetes and its care for indigenous individuals living with the disease or those involved in their care revealed three common themes: (1) mistreatment by healthcare providers; (2) mental health comorbidity; and (3) affordability of medications. All appeared to be influenced by the concepts of indigeneity, poverty, and/or gender.

### Distrust among indigenous groups and healthcare professionals

Participants held varying impressions on the quality of care that they received as patients. These ranged from positive reviews to recollections of mistreatment or abuse. These were often related to encounters in public and not private health centers, although a complete dichotomy did not exist on this factor alone. One possible reason public health centers were mentioned more often is because these centers are more readily available to the public.

A woman and her husband shared an incident involving a routine appointment for medications to manage her diabetes. She was given an intravenous injection in error, of a substance she is unable to recall, which she reports left her confined to a bed at home. Here she recalls what occurred following this event:

*“…when I got up, after two days, we went to the hospital to see, to ask what medicine they had given me. To find out why this happened to me. To know why I was in bed for two full days. But, when I got there, they told me, ‘Oh maybe because we got confused with the medicine. You weren’t supposed to get an injection.’ After I heard that, I never went back again.”*

Following this episode, she began to attend a neighboring town’s public health center to continue her diabetes management. A few months later, however, she stopped going altogether after she began to overhear “rude and offensive” remarks by some of the center’s health care professionals. Now, she sought a private clinic whenever she needed to procure prescription medications to manage her diabetes. She believed the quality of care was much higher in the latter but was quick to acknowledge that the economic costs of her medications had taken a financial toll on her household and she often went long intervals without a visit from necessity.

This narrative and others seem to detect a prevailing attitude of apathy, or at times even of antipathy, across some public health centers. For example, another participant claims that physicians purposefully prescribe insulin to diabetic patients while fully aware of the drug’s “damaging effects on the body,” particularly on processes involved in vision:

*“With time diabetes makes one blind, mainly if one is injected with insulin… It gets worse, they get worse, because insulin worsens eyesight…They must know that insulin is bad. As professionals, they must know it. Yet, they still prescribe it.”*

Individuals who held this view of insulin could not identify any practical or therapeutic use for the hormone. The professionals identified in this statement were then portrayed as people deliberately harming others. In their perspective, if patients continued to get worse while using insulin, then they tended to believe that insulin was of no benefit or even a source of potential harm. Homologous lines of thought viewing the healthcare provider as complicit or ignorant at best only further complicated the suspicious relationship between doctor and patient already present in these communities.

### Mental health comorbidities in the context of diabetes

For many members of the community, receiving the diagnosis represented a profound change in conception of the body, both physically and mentally. Nearly all participants described that the instant of becoming aware, of being informed, came with significant meaning and a change in the perception of themselves. One participant attempted to explain this metamorphosis into the sick role of a diabetic patient and the radical impact it had on his sense of self. He describes it in this way:

*“…my life practically changed. It is not the same as before. My life made a drastic turn that I never would have thought it would make… My way of seeing, my way of being. My body changed drastically. It changed everything. I am no longer the way I was before, I am not the same.”*

The participant continues by examining this transformation by noting how his family has noted drastic mood swings and unusual changes in his behavior. He himself is quick to point out that there are days when “one is happy with family and with everyone” while at other times “every little thing gets [him] upset” and “[he] only goes on arguing.” This has been so extreme, he notes, that it is beginning to affect the relationship with his family. He found these changes “disturbing.”

This participant was not alone in his concern for the effects of diabetes on his emotional state. Others also expressed sustained feelings of anxiety after having been diagnosed. Again, the refashioning of their emotional state was emphasized over any modification that may have occurred within the body itself. Several participants poignantly described this sensation. Nonetheless, it remained difficult to discern the explicit processes of how being diagnosed with diabetes altered the state of self. A woman tried to interpret the constant feeling of disquiet endured by a diabetic member of her family by proclaiming the following:

*“It makes her anxious all the time. She gets scared because she has diabetes since we have heard that diabetes, it begins to eat someone, little by little, until the person disappears from the earth.”*

Illuminated by this statement and others, diabetes can be seen as carrying large negative connotations. It carries ideas of death and decay. Many people mentioned diabetes as “entering” their bodies, almost as if an outside force had creeped inside. A man stipulated that this was due to “weak defenses” in the body which made some people more susceptible than others. While if someone’s “defenses are a little strong” then “it is not so easy for an illness to enter” after they have experienced a “susto [scare].” This then implied that there was some inherent characteristic that made some more vulnerable and others more resistant to diabetes.

When an individual in this community is diagnosed with diabetes, they begin to embody the disease along with all its implications. They state that it begins to eat away at the body and the sense of self. But, for some there is another more indirect process that also begins to occur. As mental health co-morbidities arise, adequate management of diabetes erodes until it becomes neglected. This neglect allows the disease to impose a greater deteriorating effect:

*“…she might start to get depressed. Then, she becomes unaware of her own health or doesn’t give much importance to the signs that she is presenting. After that, it affects her even more…”.*

As this woman states, people diagnosed with diabetes who also have mental health comorbidities often have outcomes which complicate treatment. This distribution of mental health comorbidities among individuals with diabetes did not seem equally distributed with respect to gender; a general consensus from participants in our sample suggested that women shared a greater piece of that burden. As one woman notes after discussing depression and loneliness for people with diabetes:

*“…what I have been talking about is more related to women in particular. Even more so if they are married.”*

Participants mentioned the notable lack of social support, especially for women. It was exceedingly common for women to be stay-at-home mothers and tend to the household. In this setting, many felt that they had few, if any, opportunities to engage in conversation with others. They confessed several times feelings of sadness, loneliness, boredom, angst, and general discontent. This was in striking opposition with that of men perspectives, where it was much more common to have feelings of camaraderie among workers and friends.

### The availability of medications

For participants with diabetes, proper access to medications was essential to manage their condition and any further complications that may arise from it. The scarce availability of medications for diabetes was a widespread problem that affected nearly every individual that we encountered. Although the country of Guatemala has universal healthcare and is required by law to provide care to all who need it, regardless of race, sex, gender, or ability to pay, this does not always occur. Hospitals, health posts, and health centers can become saturated and therefore at times unable to provide the necessary services that the public requires for both indigenous and non-indigenous populations. Medications can run out quickly due to high demand and low supply:

*“…suppose you go to the hospital, what they end up doing is that they only fill out your prescription. They give it to you and you have to go and purchase it at the pharmacy using your own money. It is supposed to be free, but it is not free. One has to be able to afford it if one can. It one can’t, well then too bad.”*

This may create disparities across the region. Although the system is intended to avoid this type of inequity, in which only certain groups are able to obtain medications, due to multiple factors it can also lead to this unintended consequence. This may lead the way towards a wider chasm between government and community, reinforcing the distrust for authority that is sometimes seen between physician and patient.

Participants were aware of inequities and often placed the blame on government-elected officials or the government as a whole. Stories of political corruption were commonplace. Narratives about politicians coercing low-income communities with food in order to procure votes were offered sometimes unsolicited. Thus, culpability for the state of affairs when discussing inequity in the national healthcare system tended to fall on either the conscious wrongdoing of individuals in power or their ineptness in navigating their respective roles. A man summarizes these two points as follows:

*“There is a lot of corruption here in Guatemala, so many people, I don’t know if you have seen in the news, are going to jail because they have stolen money from the state. There are millions of quetzals that they have taken and not correctly invested. Yea, I think that is the reason, they are stealing way too much money. That is what I think is affecting it. Either that or they are not dedicating the correct amount to the health sector, but even then, resources are still needed.”*

This participant continues by recalling how his father was affected by the inability to receive treatment due to financial instability. If the system worked as intended, he claims, the quality of life for many could be improved and/or result in reduced mortality. Therefore, participants in this study identified the issues with medication access as two-fold: medication was not always available or it was not always affordable despite the policies in place that vowed to provide all Guatemala citizens with access to free healthcare.

## Discussion

Amidst an epidemiological transition, incidence of diabetes continues to increase in rural Guatemala [4]. Here we present a setting representative of other rural impoverished communities, and we explore the indigenous community’s perceptions of diabetes and its access to care. Our data illuminated how social identity—gender, class, and race—influenced participants’ experiences with diabetes and health care. It is critical to be attune to how social categorization affects care in this region given the unique political, cultural, and historical context of an area experiencing rapid industrialization. Without understanding the cultural origins of these individuals’ experiences and perceptions we may miss how they can be uniquely affected by having health care providers they are unable to trust, mental health comorbidities, and limitations in access to medications.

The indigenous community in Guatemala has been historically discriminated against, which endangers trust in the government and by association the health care system. While violence in the past resulted from political repression following the Civil War, more recent increases in violence have been seen with the rise of socioeconomic instability coupled with political and economic news of public corruption. Indigenous individuals are now “very aware of their human and economic rights and rights as citizens of the state” and have different expectations of their governments and institutions [15]. Several accountability mechanisms have been erected to ensure that these groups are able to act on these rights, beginning with the Agreement on Identity and Rights of Indigenous Peoples in 1995 [25]. Despite gained capacity to express political opinion and open dialogue concerning inequities in recent years without retribution, indigenous individuals remain underrepresented in government and the threat of violence remains among those who monitor and respond to corruption. It would not be implausible to propose then that in a system in which indigenous populations continue to suffer from discrimination this could manifest into skeptical thoughts of that very same system. Structural violence enables and may even promote such a collective resistance to established authority [26]. In Guatemala, indigenous people have experienced similar discriminatory practices and have interactions complicated by verbal abuse, lack of access to interpreting services, and neglect in treatment [27]. Given this history, skepticism of authority seemed to be a prevailing theme across the experiences of indigenous people in our study and this mistrust appears to have a profound effect on those seeking diabetes care.

In order to improve the health of indigenous communities the Guatemalan government has enacted several policies and initiatives aimed at providing more equitable and culturally appropriate care. Among these are the Basic Service Coverage Extension Program (Programa de Extensión de Cobertura de Servicios Básicos) (1997), Law of Social Development (Ley de Desarrolo Social) (2001), and the Inclusive Health Model (Modelo Incluyente de Salud) (2011) [28]. These have sought to transform the national health system in order to improve access and provide quality care to all Guatemalan citizens regardless of social or economic status. A study that evaluated the primary health care model established by the Inclusive Health Model (Modelo Incluyente de Salud) over a 5-year period using utilization, service coverage, and quality of care metrics determined that these were significantly increased since its implementation and proved as a potential model for other nations [29]. These achievements indicate the progress and the continued efforts that are underway to ensure better access and quality care for all Guatemalan citizens.

Several explanations for how inequities have emerged beyond geographic and financial considerations also arose during some of our interviews. Class was mentioned frequently as a possible venue. Most formal education in the country, including medical education, is in Spanish. Thus, professional advancement (and class status) can be associated with the Spanish language. This language barrier can contribute not only to decreased quality in care but may also serve to further expose class divisions among indigenous populations [27]. Other indigenous populations in Latin America have expressed shame and feelings of inadequacy from being unable to speak Spanish with their health care professionals effectively, which constitutes another barrier to access [30]. Furthermore, it is important to consider how race and ethnicity play a role in healthcare disparities. Discrimination faced by indigenous populations in Guatemala can be characterized by verbal abuse, which could contribute to some individuals seeking care from traditional healers and can contribute to mistrust towards other healthcare professionals present in public and private institutions. Creating intercultural partnerships in healthcare may build trust and foster mutual learning [31]. Future studies may need to determine to what extent are indigenous groups represented in medicine and how implicit and explicit bias or the social determinants of health are presented in medical school curricula and whether these have an influence in propagating or mitigating visible disparities.

Perspectives on quality of care were also based in large part on the availability of drugs in public health centers, health posts, and hospitals. Because supply can be limited, many patients are forced to buy medications from private organizations although a free health care system is in place. Private medical centers can be seen as having more medications available and a better quality of care in Sololá but are less accessible and in many cases subject to more fees [8]. Although well-organized transportation systems have been developed in the area, particularly around Lake Atitlán, geographic location continues to play a role in accessibility for individuals who live in more rural areas away from available forms of transportation or who are unable to afford the costs needed to travel. Free and low-cost medications accessible to indigenous and non-indigenous communities can also be inconsistent [32].Thus, medication can be out of reach due a combination of limited distribution to public health centers, health posts, or hospitals and financial restraints of individuals with diabetes. There are a number of traditional healers who play a role in providing alternative forms of medications and therapies to those with diabetes who are unable to access specific medications at public or private institutions [20]. The degree of glycemic control and the effectiveness of these treatments however is unclear. In the context of diabetes care, access to medications is an essential service provided by the health care system and the disparity experienced by low-income indigenous patients appeared to only heighten the tension between different social groups. When health authorities were asked to respond to these perceptions they expressed similar concerns and a continued frustration at the systemic constraints in which they were forced to operate.

Studies suggest mental health problems remain underdiagnosed and undertreated in people with type 2 diabetes [33]. Our interviews appeared to align with these findings as it seems that indigenous people with type 2 diabetes experience mental health comorbidities tied to their community’s perception of disease. Many middle-aged participants in this study were adolescents at the time of the Civil War and the sustained traumas, anxieties, distresses or other effects compiled over the years may be presenting as deteriorating mental health today [34]. There is still much stigma surrounding mental illness within the indigenous community, yet symptoms of loneliness and depression were recognized by participants themselves or by their family members. Mental health problems appeared to be associated with changing perspectives of the sick role, changing abilities associated with type 2 diabetes complications, and status changes within the family and community. Our observations suggested that women may suffer from a higher burden of mental illness based on how gender roles are defined in the area though there may be additional contributing factors such as history of interrelationship violence and rape [35, 36]. For members of the communities in the region, men often leave the home for work throughout the day while women remain in the household. This may mean a sense of camaraderie among men co-workers but could result in little social support for women who are alone for extended periods of time. There is a higher rate of comorbid depression and diabetes in middle-aged women which can lead to issues in treatment adherence and worsening outcomes [37]. In order to mitigate some of this risk in a resource-limited country, one study has demonstrated the isolation experienced by indigenous women can begin to be remedied by psychosocial interventions which bring these women together to improve health outcomes [38]. Further studies are needed to assess what other factors may be contributing to this disparity in mental health and establish its validity; studies should examine how experiences of the Guatemalan Civil War and other historical or cultural considerations affect women [31].

### Potential targets for intervention

Designing interventions in this region with limited or narrow approaches that do not take into account the perceptions of the community and the experiences of those marginalized due to race, gender or socioeconomic status are unlikely to have significant outcomes at the population level. During observations, we witnessed moments where short lectures for school-age children on diabetes prevention and management were presented in ways that negated indigenous health beliefs and exalted Western biomedical thought in a way that could reinforce ideas of distrust between those perceived as belonging to authoritative groups and local community members. Thus, we recommend finding common dialogue when presenting information to communities regarding diabetes care. Integration of traditional healing methods with biomedical treatment has been shown to have some success in facilitating trust and improving care in Mayan populations but requires long-term collaborations that may be difficult to establish [31]. Prior studies have shown that a culturally-specific health intervention program is more likely to improve health outcomes in the region [23]. Even then, ensuring a constant stream of services and resources may be just as important. Studies in the context of HIV/AIDS in Haiti have shown that despite a divergent belief system to disease and concerns of adherence interventions that established treatments for these populations were successful [39–41]. This evidence suggests that when a group is able to observe improved health outcomes, they will attempt to continue that treatment despite personal health beliefs or shape those beliefs according to end outcomes. It is important to note though that in HIV/AIDS observable outcomes are present not long after receiving appropriate treatment [42]. Unlike HIV/AIDS, chronic diseases such as type 2 diabetes may not exhibit the same rapid and observable outcomes following initial treatment and thus this principle may be not be as applicable.

The beneficial impact of improved health outcomes on socioeconomic status has been widely studied. Equipped with the knowledge that type 2 diabetes is responsible for a high burden of disease in the region we recommend long-term partnerships with community members in order to maintain delivery programs that are reliable and consistent. Our results suggest this may in turn reduce mental health burden associated with type 2 diabetes and mitigate negative views towards healthcare professionals. Other studies have also suggested the limited screening for type 2 diabetes complications such as chronic kidney disease [43]. Given the high incidence of diabetes among indigenous populations interventions aimed at secondary and tertiary prevention are also critical. The importance of the role played by government in developing policies and programs to address indigenous health cannot be overstated as previous studies have noted [14, 44].

In addition, although the burden of diabetes in rural Guatemala appears to be high investigators must be careful to avoid creating or further reinforcing stigmas that may present for individuals with diabetes. In this same realm one must also consider the target population of directed interventions. Medicalization of diabetes and expanding the social construct of health to include people with pre-diabetes only further assigns the sick role to a greater number of individuals who may not otherwise be in any real danger of deteriorating their current or future quality of life given they receive proper care. As our results suggest, such categorization into the sick role may have a significant impact on mental health and may at least be partially avoided by conducting careful screenings and seeking thoughtful and honest insight on how a diagnosis may impact a patient given their race, gender, and/or socioeconomic status. Finally, genetics and the “susceptibility” of diabetes among indigenous groups must also be questioned and assessed in order to reduce the possibility of characterization, prejudice, and stigma. We recommend that future interventions should be developed, implemented, and conducted in collaboration with local indigenous communities to mitigate the burden of type 2 diabetes across the region. As has been observed, interventions may be hailed as successful when they qualitatively appear to reduce burden of a single disease or improve specific health outcomes while unintended consequences persist resulting in inequities elsewhere [45].

### Limitations

This study contains some limitations. First, we did not collect interview data from healthcare professionals. This could have provided a different perspective on how social, economic, cultural, and historical factors affect diabetes care and support or refute some of the claims made here. We have attempted to include shared views of healthcare professionals when possible. Second, two participants included in this study did not speak Spanish, but rather Tz’utujil, a Mayan-derived language. An intermediary served as an interpreter who was also a healthcare professional in the local community hospital. They were instructed to render participants’ statements as faithfully as possible and we have reasonable expectations that this was done, but it is otherwise impossible to know for certain what may have been missed or misunderstood in this initial encounter as we do not speak Tz’utujil. The fact that the authors are not fluent in the local spoken language of the towns provided further challenges. It is possible that incomplete information could have been collected or misinterpreted from interviews in Spanish given that a second language may have been spoken more frequently among some of the participants who participated in this study. We attempted to mitigate this by offering the option to conduct the interview in the local language through an interpreter. However, given that interpreters were from the local area could have resulted in some hesitation among participants. Additionally, concerns arose during the study design phase regarding how the author’s identity and background might influence data collection and analysis. Not identifying as indigenous nor being native to the region it was unclear how this might impact participant response. However, it appeared that being a native Spanish speaker and a foreign national also from Latin America presented an opportunity for participants to openly express their beliefs, opinions, and frustrations with fewer technical language barriers. In some ways having little association with government or private healthcare entities created an opportunity to serve as a messenger to inform local officials regarding current community opinion without jeopardy to participants as all data remained anonymous. Despite these challenges, we believe that the data and the results presented here do provide an accurate account of the perceptions held by local community members and their interaction with others.

## Conclusion

Rising rates of diabetes pose potential health risks for indigenous communities in Guatemala. Through a modified grounded theory approach, hours of participant observations and over a dozen interviews conducted with local community members, several main themes emerge. Interviews suggest that indigenous people experience mistrust in the health care system, unreliable access to care, and mental health comorbidity in the context of type 2 diabetes care. Perspectives of participants appear to be influenced by historical events, perceptions of political and national economic stability, and prior personal experiences with healthcare all of which may in some part contribute to mistrust of individuals perceived as belonging to groups of authority. It is also observed that perceptions of class, gender, and race influence the experience of diabetes care within the established healthcare system for indigenous individuals. Taken together, historical, societal, and political context needs to be taken into account when endeavoring to provide care to indigenous patients with diabetes in Guatemala. Ultimately, future interventions should consider the complex interplay between social, cultural, and historical dynamics impacting health outcomes in order to produce context-appropriate strategies that will help mitigate the burden of type 2 diabetes that the region faces.

## Data Availability

The transcripts used to support the conclusions of this article are available in English and Spanish upon reasonable request.
